# A Comparison of Two Peripheral Nerve Blocks Combined With General Anesthesia in Elderly Patients Undergoing Arthroplasty for Hip Fractures: A Pilot Randomized Controlled Trial

**DOI:** 10.3389/fsurg.2022.715422

**Published:** 2022-02-17

**Authors:** Qingfu Zhang, Ming Ling, Xintao Wang, Derong Cui

**Affiliations:** ^1^Department of Anesthesiology, Shanghai Jiaotong University Affiliated Sixth People's Hospital, Shanghai, China; ^2^Department of Orthopedics, Huadong Hospital Affiliated to Fudan University, Shanghai, China

**Keywords:** peripheral nerve blocks, general anesthesia (GA), combined anesthesia, hip fracture, hemodynamics

## Abstract

**Background:**

Combined anesthesia can be a promising option for hip surgery when neuraxial anesthesia is contraindicated. Lumbar and sacral plexus blocks, and femoral nerve and lateral femoral cutaneous (LFC) nerve blocks in combination with general anesthesia (GA) are commonly used in elderly patients undergoing arthroplasty for hip fracture surgery. However, no study has compared these two anesthetic strategies in the perioperative period.

**Methods:**

A total of 41 elderly patients scheduled for arthroplasty for hip fracture surgery were randomized into group A (*n* = 20) and group B (*n* = 21). Group A received femoral nerve block, LFC nerve blocks, and GA, and group B received lumbar plexus block, sacral plexus block, and GA. Primary outcomes were incidences of hemodynamic events and changes in blood pressure (BP) and heart rate (HR). Secondary outcomes included time and drug consumption, infusion and bleeding volume, eyes opening time after surgery, and postoperative quality recovery rate.

**Results:**

Compared with group B, group A showed a lower incidence of intraoperative hypotension (*p* < 0.001), higher BP [including mean arterial pressure (MAP), systolic BP (SBP), and diastolic BP (DBP)] following induction (IN), and higher HR from mid-surgery. Time required for nerve blockade (*p* < 0.001) and ephedrine consumption was significantly shorter in group A (*p* < 0.001), while sufentanil consumption was higher as compared to group B (*p* = 0.002). No significant differences in other intraoperative parameters and postoperative quality recovery rate were reported during the observation.

**Conclusion:**

Our pilot data indicate that compared with lumbar and sacral plexus blocks, femoral nerve and LFC nerve blocks may provide more stable intraoperative hemodynamics and a comparable postoperative recovery for elderly patients undergoing arthroplasty for hip fracture under GA. Further studies with a larger sample size are needed to derive stronger evidence.

## Background

Hip fractures are one of the most frequent fractures in elder adults, leading to pain, functional impairment, and socioeconomic burden, and affecting millions annually with 20–30% 1-year mortality (1–3). Global aging, together with recent advances in public health, is predicted to increase the number of hip fracture cases by 2% per year over the next few decades (4). Surgical treatment involving internal fixation and arthroplasty is considered as the best option for patients with hip fracture, especially the elderly, and allow early ambulation, which reduces fracture-related complications (5). However, studies show that patients who undergo hip fracture surgery have an increasing chance of accompanying multiple comorbidities that may complicate anesthesia and analgesia for these patients (4).

Satisfactory anesthesia is patient-specific as well as anesthesiologist-specific, and the ideal treatment technique for elderly patients with hip fracture remains controversial. Both general anesthesia (GA) and neuraxial anesthesia have been widely used till date. However, meta-analysis studies have failed to demonstrate a significant difference in outcomes with the two methods (6). While neuraxial anesthesia is preferred for majority patients, it may not be routinely used especially in elderly patients under anticoagulant therapy. A recent survey of anesthetists has indicated variability of perioperative anticoagulant management in patients undergoing neuraxial anesthesia with anesthetists unwilling to proceed with the procedure or preferring the use of GA (7). Therefore, in elderly patients, GA and regional anesthesia (RA) are commonly used as alternatives to neuraxial anesthesia. Studies report that in some cases, GA or RA alone do not achieve satisfactory anesthesia, while a combination of GA and RA shows better results (8, 9). The advantages of combined GA and RA include earlier return of function as it relies mainly on the RA, thus reducing the amount of general anesthetic delivered to the patient (10). Due to the complex sensory innervation of the hip joint, no single nerve block allows sufficient analgesia and no single injection allows all the sensory nerves to be reached (11). Thus, the combination of multiple peripheral nerve blocks (PNBs) plays an essential role in using RA alone or in combination with GA.

Several different types of PNBs, namely the femoral nerve block, lateral femoral cutaneous (LFC) nerve block, lumbar plexus, and sacral plexus blocks, have been used during hip surgeries (8, 9, 12). A large body of evidence suggests that a combination of lumbar and sacral plexus block or the blockade of femoral nerve and LFC nerve can provide effective anesthesia during hip surgeries (9, 13–17). A recent Cochrane review (18) indicates that the femoral nerve and LFC blocks have been largely popular among anesthetists. However, there is a need for comparative studies of different PNB techniques to establish the superiority of one approach over the other. Despite intense research on different PNBs for hip surgeries, there is limited comparative evidence between femoral nerve and LFC blocks vs. lumbosacral plexus blocks. The main goal of the current study is to compare the blockade of femoral nerve and LFC nerve plus GA vs. the blockade of lumbar plexus, and sacral plexus plus GA on intraoperative hemodynamics and short-term postoperative recovery.

## Materials and Methods

The trial was registered at ClinicalTrials.gov (NCT02635763) on December 17, 2015. The study was conducted at the Department of Anesthesiology, Shanghai Sixth People's Hospital from December 2015 to October 2017 with the approval from the Institutional Ethical Committee of Shanghai Sixth People's Hospital (No. 2015-35-1).

### Study Population

Patients scheduled for surgery due to acute hip fracture were eligible for this study, and the inclusion criteria were as follows: (1) patients are from 65 to 85 years old; (2) the American Society of Anesthesiologists (ASA) physical status I–III; (3) body mass index (BMI) <30 kg/m^2^; (4) the Mini-Mental State Examination > 23; and (5) patients who undergo arthroplasty or internal fixation. The exclusion criteria were as follows: (1) serious systemic diseases, including respiratory dysfunction, cardiac, and renal insufficiency; (2) a history of cognitive function disorder or mental illness; (3) a history of cerebral infarction and related sequela; (4) blood coagulation dysfunction; (5) hearing disorder or visual impairment; (6) unable to complete the assessment; (7) surgical duration > 90 min; and (8) bleeding volume > 1,000 ml.

However, after the trial began, we found that there was a significant difference in the duration of surgery and pain stimulation between patients undergoing arthroplasty and those undergoing internal fixation, and therefore the trial was modified to enroll patients who underwent arthroplasty only.

### Randomization and Allocation Concealment

A research assistant who was not involved in the study prepared the randomization table using a computer-generated list. Randomization method was single and the trial was parallel in nature. Group allocation was then enclosed into sequentially numbered and sealed envelopes. Each patient was assigned a sequence number according to the time of enrolment. Before anesthesia, only the anesthesiologist had access to the envelope in accordance to the patient's sequence number and the patient was allocated into either group A or B in a ratio of 1:1. The intervention was blinded to the investigators who were in charge of data collection or clinical assessment, while it was not blinded to the patients and anesthesiologist.

### Anesthetic Protocol

Group A received femoral nerve block, LFC nerve blocks, and GA while group B received lumbar plexus block, sacral plexus block, and GA. To perform femoral nerve and LFC nerve blocks, the patients were placed in the supine position. In the inguinal region, the femoral nerve was identified lateral to femoral artery, followed by an injection of 15 ml 0.5% ropivacaine, and the LFC nerve was identified superficial to sartorius muscle, followed by an injection of 5 ml 0.5% ropivacaine (19, 20). The approach of posterior was used to perform lumbar and sacral plexus blocks. Specifically, the patients were placed in the lateral position with hips flexed and the side to be blocked uppermost. Lumbar plexus was identified at L3 level (caudad to transverse process and 1–1.5 cm anterior to its surface) and at L3–L4 levels (in the space of psoas major), followed by an injection of 12.5 ml 0.5% ropivacaine at each of the two sites (21). Sacral plexus was identified superficial to piriformis and injected with 15 ml 0.5% ropivacaine (21). The PNBs were performed under ultrasound guidance. After PNBs, the evaluation of sensation and movement of the blocked side was conducted for 5, 10, and 15 min, and the adequacy of PNBs was assessed at the final evaluations. To avoid interference with cognitive function, no sedation was applied during PNBs.

Combined GA was performed after the adequacy of PNBs was confirmed. All the patients received an infusion of hydroxyethyl starch 130/0.4 (5 ml/kg) 15 min before induction (IN) to prevent hypotension. Then GA was induced with 0.5 μg sufentanil and 1.5–2 mg/kg propofol, followed by laryngeal mask airway (LMA) and mechanical ventilation, and GA was maintained with the inhalation of sevoflurane (0.7–1.2 MAC) and 100% oxygen. The inhalation of sevoflurane was adjusted, according to clinical judgment and a target bispectral index (BIS) range of 40–60. Intraoperative autonomic respiration was maintained after the restoration of spontaneous breathing. Supplemental analgesia (1–5 μg sufentanil) was administered if required to control the respiratory rate at 10–20/min.

The anesthesia of both groups was conducted by the same senior anesthesiologist, and the related parameters were recorded by an independent anesthesiologist assistant. All the surgeries were conducted by the same group of surgeons in the morning. During the whole course in the OR, arterial blood pressure (BP) was monitored by radial artery cannulation. Severe hypotension was treated with an injection of 5 mg ephedrine every 3 min until the systolic BP (SBP) was restored. Sinus bradycardia was treated with 0.5 mg atropine.

Postoperative analgesia included a 3-day intravenous analgesia (tramadol 10 mg/h and lornoxicam 0.32 mg/h) and a supplemental injection of parecoxib (40 mg/12 h if required).

### Assessment of Postoperative Recovery

The Postoperative Quality Recovery Scale (PQRS) was used for the assessment of postoperative recovery. Baseline (BL) assessment was conducted a day before the surgery. The time 0 was defined as following the last skin stitch, and postoperative assessment was conducted at 15 min, 40 min, 1 day, and 3 days after the surgery. Among the domains of PQRS, the assessment of activities of daily living (ADL) was not applicable for patients with hip fracture, leaving five domains (physiologic, nociceptive, emotive, cognitive, and overall patient perspective) for the assessment. The assessment was performed by an independent investigator who was blinded to the allocation.

Recovery was defined as “return to BL values or better”: if the scores of a certain item were greater than the BL value, the recovery was scored.

### Outcomes and Measurements

Primary outcomes of the study included intraoperative hemodynamics: incidence of hemodynamic events (hypertension, hypotension, and sinus bradycardia) and a variation of mean arterial pressure (MAP), SBP, diastolic BP (DBP), and heart rate (HR). MAP, SBP, DBP, and HR were recorded at the following time points: a day before the surgery (BL), following PNBs, following IN, 5 min after beginning (S5) of surgery, 25 min after beginning (S25) (as mid-surgery), before skin closure (C-bef) and after skin closure (C-aft).

Secondary outcomes included time consumption (PNBs, GA, and surgery duration), intraoperative drug consumption (sufentanil and ephedrine), infusion and bleeding volume, eyes opening time after surgery, and PQRS recovery rate at the recorded time point.

Hypotension and hypertension were defined, respectively, as a drop or an increase of more than 30% in SBP compared with BL. Bradycardia was defined as HR <45 bpm.

### Power Analysis

The reported incidence of intraoperative hypotension in hip surgery varied from study to study. Intraoperative hypotension occurred in more than half patients and was more often during GA than spinal anesthesia (22). The incidence of intraoperative hypotension following combined anesthesia in elderly patients with hip fracture was rarely reported. According to the observation, about 70% of patients who received lumbar and sacral plexus blocks plus GA in our department suffered from intraoperative hypotension and the incidence was markedly reduced to <30% with the use of femoral nerve and LFC nerve blocks plus GA. Thus, a sample size of 21 patients for each group would have ~80% power to detect such a difference on hypotension incidence. A sample size of 30 for each group was set to evaluate the excluded cases and the follow-up loss.

### Statistical Analysis

Continuous variables were presented as mean ± SD if normally distributed, or as median and interquartile range otherwise. Categorical variables were presented as frequencies and percentage. Between-group comparisons were conducted using a *t*-test for continuous variables and χ^2^/Fisher's exact test for categorical variables. A mixed-effects model was used to compare the repeated measurements at different time points. Stata 13.0 (StataCorp, College Station, TX, USA) was used for the analysis, and the value of *p* < 0.05 was considered as statistically significant.

## Results

### Demographic Information

**Figure 1:**
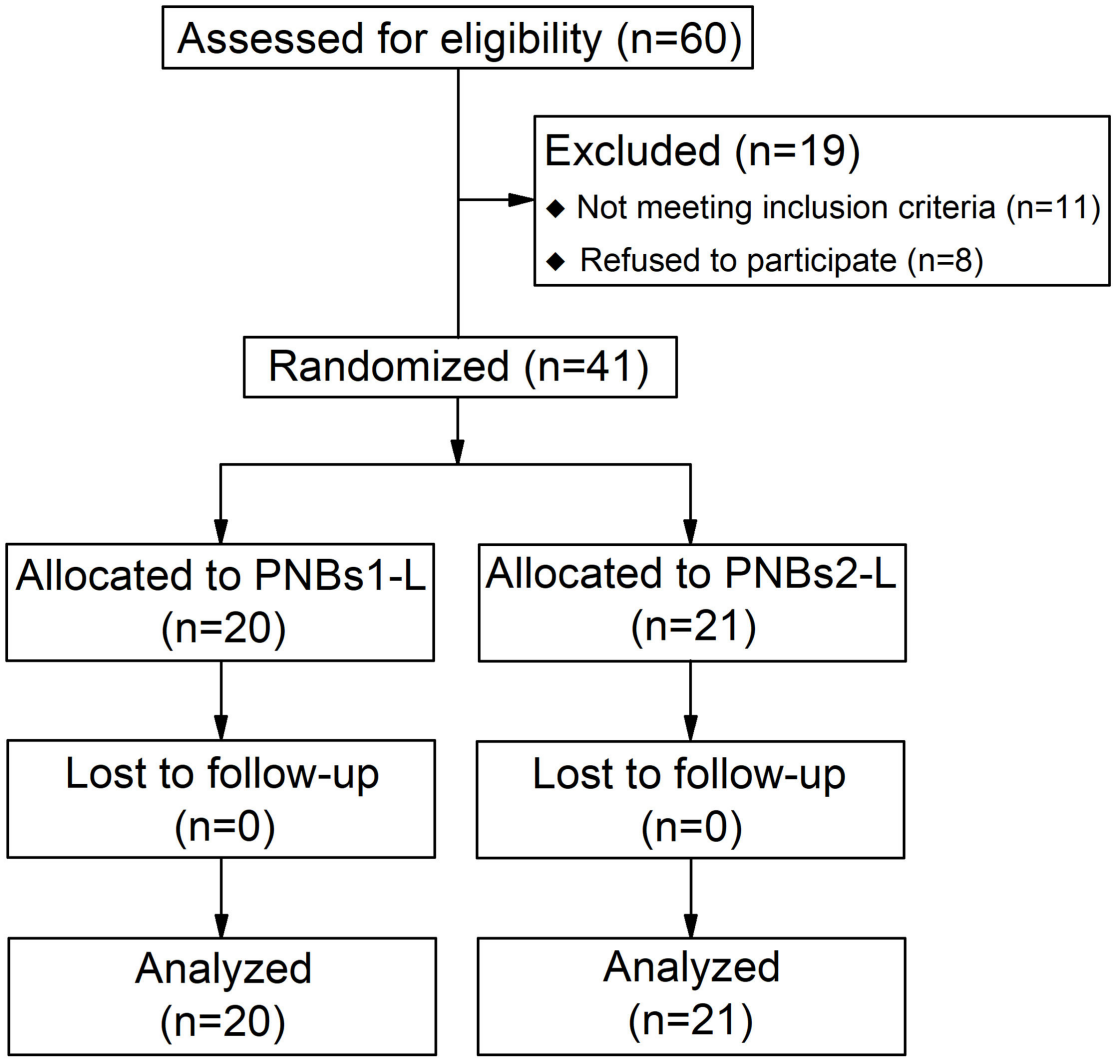
Consolidated standards of reporting trials (CONSORT) flow diagram of patient group enrolment.

A total of 60 consecutive patients were initially recruited in this study. Of them, 19 patients were excluded (11 failed the inclusion criteria and eight were rejected). Among the remaining 41 patients, 20 were randomized into group A and 21 were randomized into group B, and both groups were under observation during the consequent short-term follow-up. Considering the small number of patients potentially recruited for this study, the trial should be considered as a pilot RCT. The Consolidated Standards of Reporting Trials (CONSORT) flow is shown in Figure 1.

The mean age of the subjects was 77.83 ± 6.27 years with female patients being the majority (26/41). Between-group comparison revealed no significant differences in demographic data, such as sex, age, height, weight, BMI, years of education, injured side, fracture type, and ASA grade (Table 1).

**Table 1:** Subject demographics.

	**Group A (*n* = 20)**	**Group B (*n* = 21)**	***P*-value**
Sex, male/female	6 (30%) / 14 (70%)	9 (43%) / 12 (57%)	0.520
Age, years	78.1 ± 5.64	77.57 ± 7.08	0.793
Hight, cm	159.2 ± 7.50	161.85 ± 6.88	0.244
Weight, kg	58.2 ± 9.70	59.80 ± 11.57	0.633
BMI, kg/m^2^	22.92 ± 3.19	22.71 ± 3.49	0.841
Years of education	9.25 ± 0.375	8.80 ± 3.84	0.712
Injured side, left/right	10 (50%)/10 (50%)	11 (52%)/10 (48%)	1.000
Type of fracture, neck/trochanteric	13 (65%)/7 (35%)	15 (71%)/6 (29%)	0.744
ASA grade, I/II/III	1 (5%)/15 (75%)/4 (20%)	1 (5%)/17 (81%)/3 (14%)	0.698

*Continuous variables are marked as mean ± SD. Categorical variables are shown as frequencies and percentage*.

*Group A: femoral nerve and lateral cutaneous nerve blocks plus general anesthesia (GA); Group B: lumbar plexus and sacral plexus blocks plus GA*.

*BMI, body mass index; ASA, American society of anesthesiologists*.

### Intraoperative Hemodynamics

The overall outcome data of the subjects are summarized in Table 2. In terms of intraoperative hemodynamics, patients in group B had a greater incidence of hypotension as compared to group A (71 vs. 15%, *p* < 0.001). Hypertension was more prevalent in group A (25 vs. 10%) and sinus bradycardia was more prevalent in group B (67 vs. 50%), although there was no statistical significant difference between the two groups for either variables.

**Table 2:** Intraoperative data of the subjects.

	**Group A (*n* = 20)**	**Group B (*n* = 21)**	***P* value**
Time required for block/min	6.40 ± 1.50	21.20 ± 3.40	<0.001
Anesthesia duration/min	60.25 ± 16.58	67.38 ± 22.56	0.257
Surgery duration/ min	48 ± 13.31	53.57 ± 13.52	0.191
Hypertension incidence	5 (25%)	2 (10%)	0.238
Sinus bradycardia incidence	10 (50%)	14 (67%)	0.350
Hypotension incidence	3 (15%)	15 (71%)	<0.001
Ephedrine consumption/mg	2.5 (0,5)	7.5 (0,20)	<0.001
Sufentanil consumption/ug	8.37 ± 2.95	5.11 ± 3.30	0.002
Transfusion volume/ml	1,100 (1,000, 1,200)	1,200 (1,000, 1,450)	0.196
Bleeding volume/ml	353.50 ± 139.10	298.09 ± 147.73	0.224
Eyes opening time after surgery/ min	10.75 ± 4.12	12.33 ± 6.39	0.354

*Continuous variables are marked as mean ± SD, or median and interquartile range. Categorical variables are shown as frequencies and percentage*.

*Group A: femoral nerve and lateral cutaneous nerve blocks plus GA; Group B: lumbar plexus and sacral plexus blocks plus GA*.

**Figure 2:**
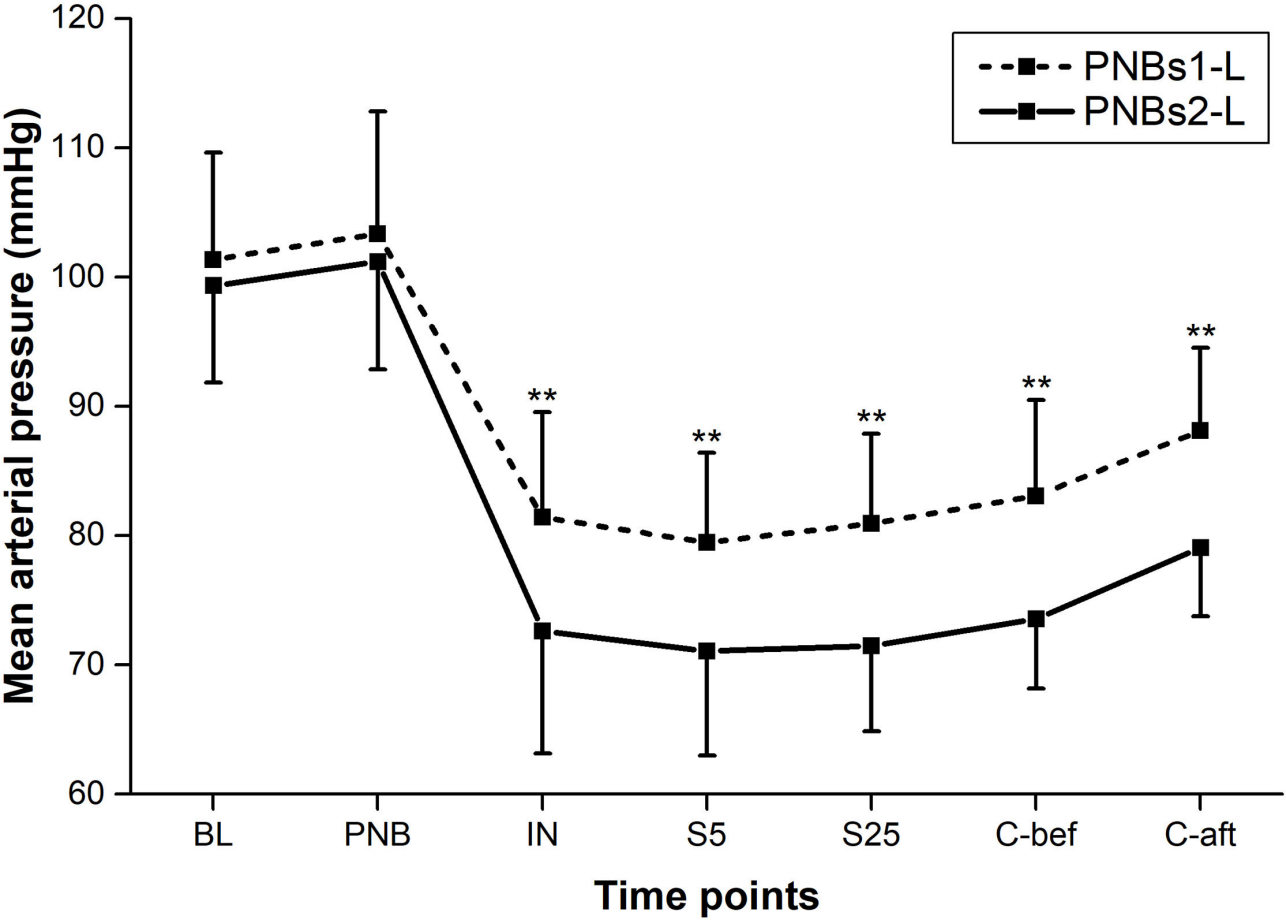
Mean arterial pressure (MAP) at each time point of perioperative period. Between-group comparison: **p < 0.01.

**Figure 3:**
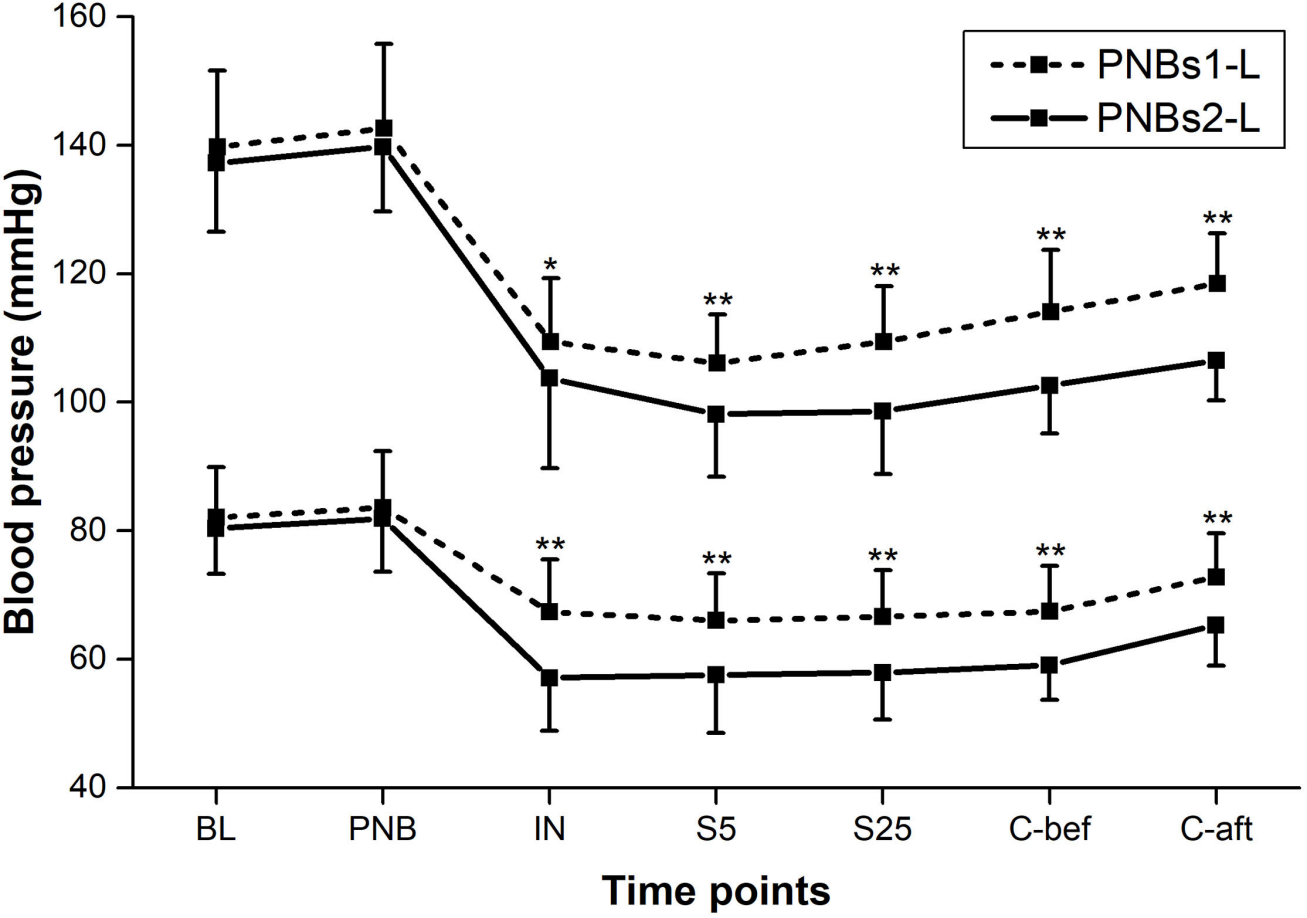
Systolic blood pressure (SBP) and diastolic blood pressure (DBP) at each time point of perioperative period. Between-group comparison: *p < 0.05, **p < 0.01.

**Figure 4:**
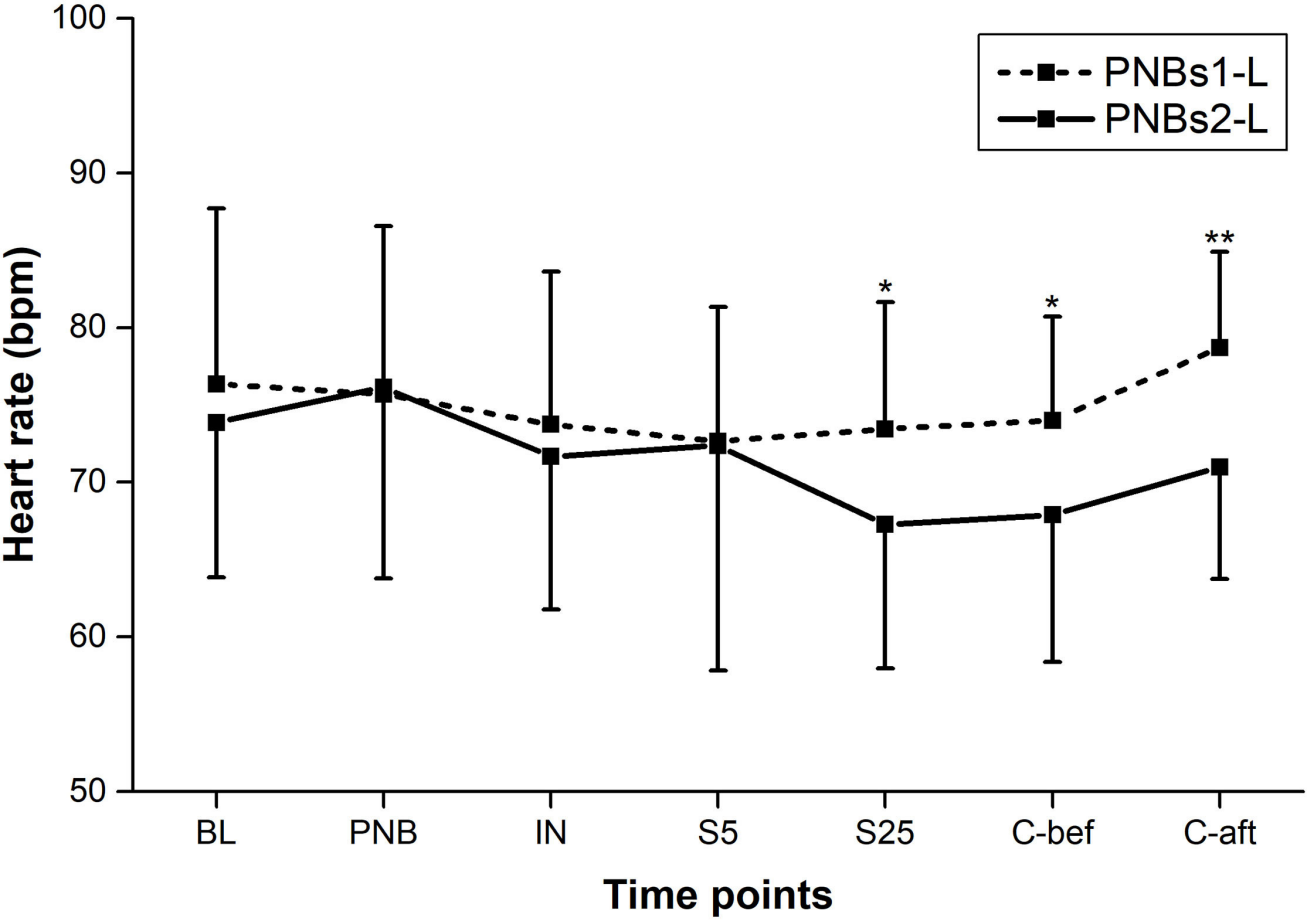
Heart rate (HR) at each time point of perioperative period. Between-group comparison: *p < 0.05, **p < 0.01.

All BP parameters, including MAP, SBP, and DBP, showed similar dynamics in both groups; markedly declining following IN and rising gradually from mid-surgery (Figures 2, 3). From IN to skin closure, the BP was significantly higher in group A than in group B (*p* < 0.01, except *p* < 0.05 for DBP following IN) (Figures 2, 3). Similarly, HR was significantly higher in group A as compared to group B from mid-surgery (*p* < 0.05, except *p* < 0.01 after skin closure) (Figure 4).

### Secondary Outcomes

Patients in group A required significantly less time for a nerve block as compared to group B (6.4 ± 1.5 vs. 21.2 ± 3.4 min, *p* < 0.001). No significant differences were found in the anesthesia or surgery durations. Patients in group B were administered more ephedrine than patients in group B (median, 7.5 vs. 2.5 mg, *p* < 0.001), due to a higher incidence of hypotension in that group. Sufentanil consumption was also significantly increased in group A compared to group B (8.37 ± 2.95 vs. 5.11 ± 3.30 mg, *p* = 0.002). Both groups showed no significant differences on other parameters, including transfusion and bleeding volume, and eyes opening time after surgery. No significant differences were found between the two groups in the postoperative recovery domains, such as physiology, emotion, nociception, and cognition at time points of T15, T40, D1, and D3, and all of the patients were satisfied or completely satisfied with the anesthesia treatment.

## Discussion

This study compared two combined anesthesia strategies on intraoperative hemodynamics and short-term postoperative recovery among elderly patients who undergo hip fracture surgery. We reported that femoral nerve and LFC nerve blocks plus GA demonstrated more stable intraoperative hemodynamics characterized by higher BP and fewer hypotension, and both strategies achieved similar outcomes in short-term postoperative recovery.

Although the use of the femoral nerve and LFC nerve blocks alone is not sufficient in hip fracture surgery, the sensory innervation region of the two nerves overlaps largely with the surgical region. The hip joint is innervated by multiple sensory nerves. Superficially, femoral nerve and LFC nerve innervate the anterior and lateral thigh, which meets the need of hip surgery incision in most cases. In a deep layer, the hip joint is innervated by the articular branches of obturator, femoral, sciatic, and gluteal nerves, so the blockade of femoral nerve provides some analgesia for the hip. Addition of GA enhances analgesic effect of PNBs and allows patients to profit from the potential hemodynamic advantages of PNBs vs. neuraxial or GA (23, 24). A conventionally recommended method, neuraxial anesthesia, is associated with potential risks in elderly patients (25). Indistinct advantage in reducing complications in addition to higher technical requirements and uncomfortable experience makes neuraxial anesthesia less preferable in this age group. Our results indicated that femoral nerve and LFC nerve blocks elevated the overall intraoperative BP, which contributed to the reduction of hypotension. Although elevated intraoperative BP may lead to excessive bleeding, the bleeding volume of group A was not significantly greater, indicating that the elevation of BP was within a normal range. The blockade of lumber plexus would inevitably block the sympathetic nervous system within this area, leading to a reduction in effective circulating blood volume, explaining the observed prevalence of hypotension in group B. Additionally, inadequately positioned injection or the dispersion of local anesthetic into the epidural space may also increase the risk of hypotension (26–28). Compared with plexus block, femoral nerve and LFC nerve blocks target more peripheral nerves, allowing for more accurate analgesia, while causing less complications.

Currently, there are only a few studies that investigate the incidence of hypotension associated with the use of combined anesthesia. Chen et al. (29) conducted a comparison between combined anesthesia (lumbar plexus and sciatic block plus LMA) and GA (endotracheal intubation) and found that the former resulted in a lower incidence of intraoperative hypotension. However, the reported incidence of Chen's study was much lower than this study, which may be explained by the differences in study population and measuring technique. Other studies did not report the incidence of hypotension, while pointing out the benefits of combined anesthesia. Duarte et al. (30) found that both lumbar plexus block and epidural lumbar block were not associated with hemodynamic instability when combined with GA. Mei et al. (15) found that a lumbosacral plexus block combined with GA reduced the need for opioids, offered satisfactory postoperative analgesia, and led to better postoperative outcomes in combination with light sedation. Moreover, the combination of PNBs and GA was associated with the reduced requirement of systemic anesthetic and muscle relaxants, which leads to a more rapid recovery of the patients (29, 31). In our study, while group A was administered with more sufentanil, there was no significant increase in hypotension incidences, probably due to the moderate hemodynamic impact of sufentanil.

Both groups exhibited similar postoperative recovery as indicated by the measured domain of PQRS. These results indicate that the two combined anesthesia regiments had a similar influence on a short-term recovery. While the choice of anesthesia does not impact mortality rates, choice of adequate anesthesia may prevent possible complications. A few studies show that intraoperative hypotension was associated with acute kidney injury, ischemic stroke, and myocardial injury (2, 32–36). Thus, it is hypothesized that femoral nerve and LFC nerve blocks plus GA may reduce the risk of these complications by appropriately perfusing the important organs.

In clinical practice, anesthesia techniques that require a lateral position are difficult to conduct in some cases, especially when some patients could not stand the severe pain caused by this position. As femoral nerve and LFC nerve blocks do not require the change of body positions, these methods of anesthesia are favored both by the patients and by anesthesiologists. Although PNBs are not as efficient as neuraxial anesthesia for muscle relaxation, this limitation of PNBs may be less concerning in geriatric patients that usually do not require a thorough muscle relaxation due to their lower muscular tension.

The major limitation of the current study is a relatively small sample size. Despite initially recruiting 60 patients, the final analysis could include just 41 patients. Hence, our trial should be considered as a pilot RCT, the results of which need to be corroborated with future large-scale studies. Secondly, there was a lack of control group in our study. We only compared the two different RA techniques in combination with GA and there was no control group of only nerve blocks or only GA. Addition of such groups could have improved the quality of evidence. Thirdly, there were differences in the volume of anesthetic agents used in our study, which could have skewed the hemodynamic results. Lastly, we only compared immediate outcomes of the two anesthesia techniques and a long-term follow-up was missing.

## Conclusion

In conclusion, our results of our pilot study suggest that lumbar and sacral plexus blocks in combination with GA may be superior to femoral nerve and LFC nerve blocks plus GA for elderly patients undergoing arthroplasty for hip fracture surgery. The use of lumbar and sacral plexus blocks vs. femoral nerve and LFC nerve blocks could result in more stable hemodynamics with comparable postoperative recovery. Further research is needed to validate the results in a larger population.

## Data Availability Statement

The raw data supporting the conclusions of this article will be made available by the authors, without undue reservation.

## Ethics Statement

The studies involving human participants were reviewed and approved by Institutional Ethical Committee of Shanghai Sixth People's Hospital (No. 2015-35-1). The patients/participants provided their written informed consent to participate in this study.

## Author Contributions

QZ designed the project and prepared the manuscript. ML, XW, and DC were involved in data collection and data analysis. DC edited the manuscript. All authors read and approved the final manuscript.

## Funding

This work was funded by the National Natural Science Foundation of China (Grant No. 81974284).

## Conflict of Interest

The authors declare that the research was conducted in the absence of any commercial or financial relationships that could be construed as a potential conflict of interest.

## Publisher's Note

All claims expressed in this article are solely those of the authors and do not necessarily represent those of their affiliated organizations, or those of the publisher, the editors and the reviewers. Any product that may be evaluated in this article, or claim that may be made by its manufacturer, is not guaranteed or endorsed by the publisher.
